# Supplementary material for: Neural mechanisms underlying the effects of physical fatigue on effort-based choice
Source: Nat Commun. 2020 Aug 12;11:4026. doi: 10.1038/s41467-020-17855-5 (PMC7424567; doi:10.1038/s41467-020-17855-5)
Supplement: Supplementary file 1 — Supplementary Information [file 41467_2020_17855_MOESM1_ESM.pdf]

Supplementary Information for:

Neural mechanisms underlying the effects of physical fatigue on effort-based choice

Patrick S. Hogan[1], Steven X. Chen[1], Wen Wen Teh[1], and Vikram S. Chib[1,2,3*]

[1]Department of Biomedical Engineering, Johns Hopkins School of Medicine, Baltimore, MD 21205

[2]Kavli Neuroscience Discovery Institute, Johns Hopkins University, Baltimore, MD 21205

[3]Kennedy Krieger Institute, 707 North Broadway, Baltimore, MD 21205

*Correspondence and requests for materials should be addressed to:

Vikram S. Chib
707 North Broadway
Baltimore, MD 21205, USA
443-923-2716
vchib@jhu.edu

SUPPLEMENTARY FIGURES

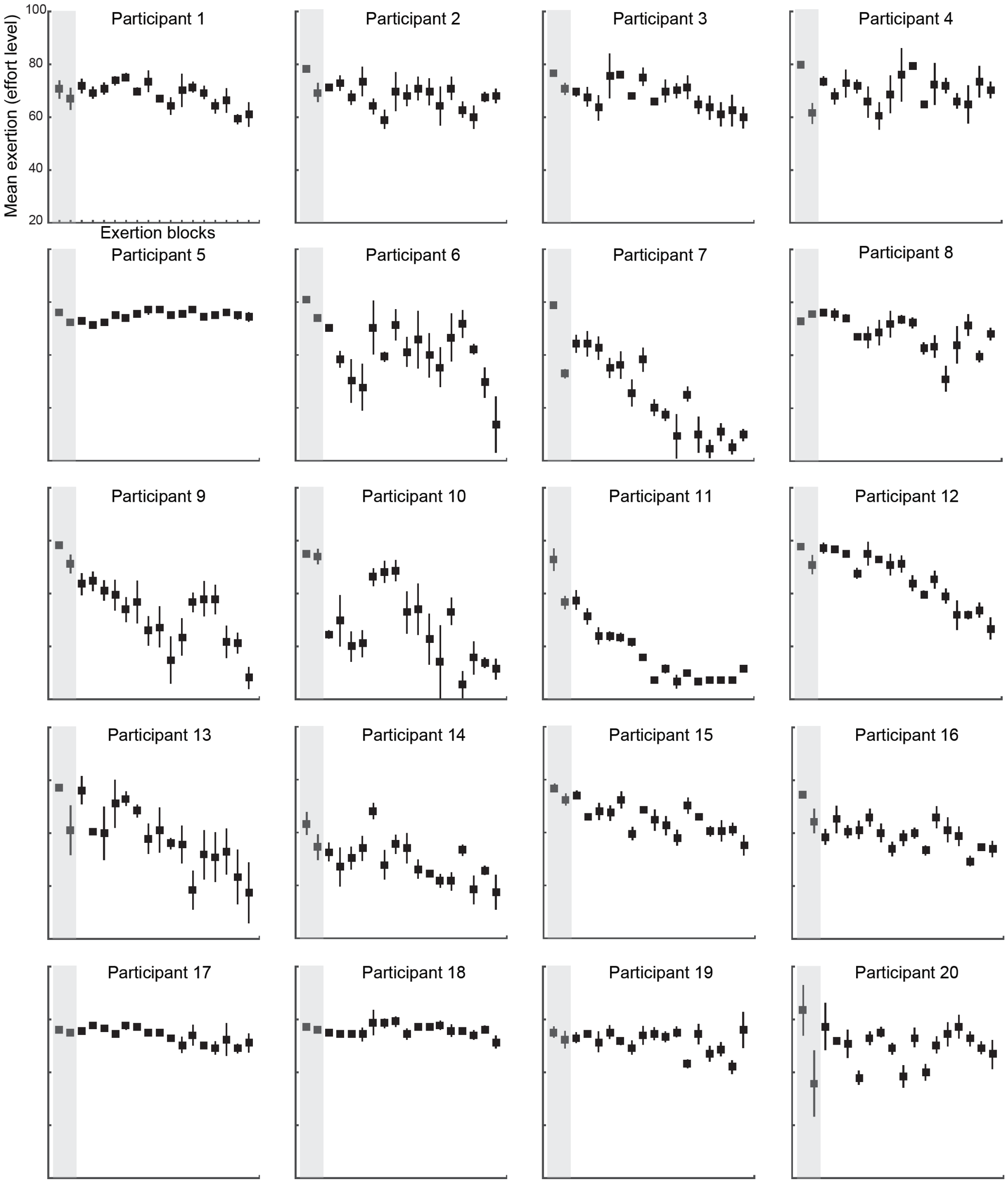

Supplementary Figure 1 (n = 20).

Mean exertion during the Fatigue Choice Phase for individual participants. Mean exertion is presented in units of effort. For the initial exertion block (shaded gray), the mean exertion for the first and last five trials are shown. For subsequent blocks, the mean exertion over the full exertion block is shown. Error bars indicate SEM.

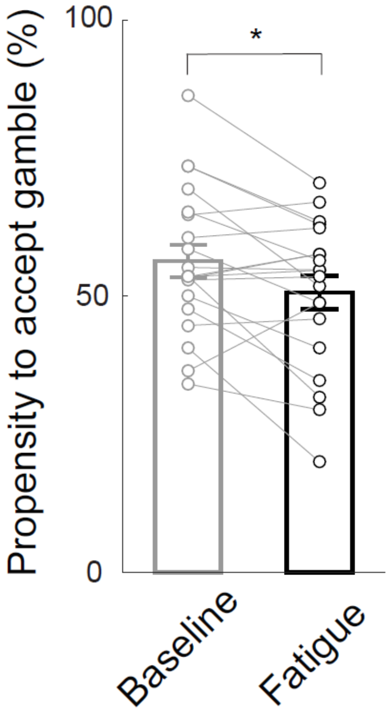

Supplementary Figure 2 (n = 20).

Effort gamble acceptance rate between Baseline and Fatigue Choice Phases. We observed a statistically significant decrease in the percentage of effort gamble choices made between the Fatigue and Baseline Choice Phases (mean change: 5.73% (SD = 9.38%), two-tailed paired sample $t_{19} = 2.73$, $p = 0.01$); on average participants choose the risky option less frequently when fatigued. Error bars indicate SEM. *$p < 0.05$.

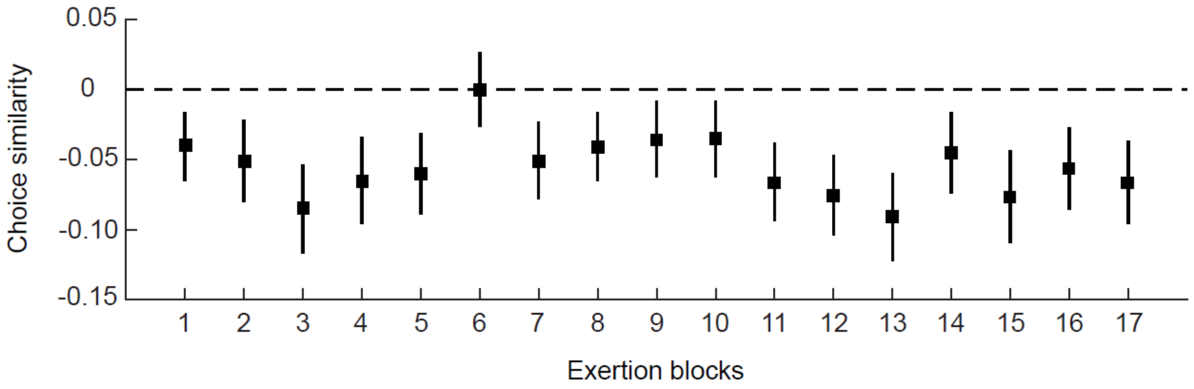

Supplementary Figure 3 (n = 20).

Choice similarity metric over the course of the Fatigue Choice Phase in the main experiment. The plot shows mean choice similarity (see Supplementary Methods for details) across participants for each of the 17 choice blocks after the initial fatiguing exertion block. On average, participants' choice similarity metric was negative (mean choice similarity: -0.06 (SD = 0.02); two-tailed one-sample t-test against the null hypothesis that choice similarity is zero: $t_{16} = -10.32$, $p < 0.001$), indicative of more risk averse decision-making, consistent with what we observed in our primary effort utility analysis. Notably, there was not a significant effect of exertion block on choice similarity (results of a general linear model on the effect of exertion block on choice similarity: $\beta_{Block} = -0.001$, $p = 0.54$) suggesting that after the initial bout of physical fatigue, participants' change in subjective preference remains relatively constant throughout the Fatigue Choice Phase. Error bars indicate SEM.

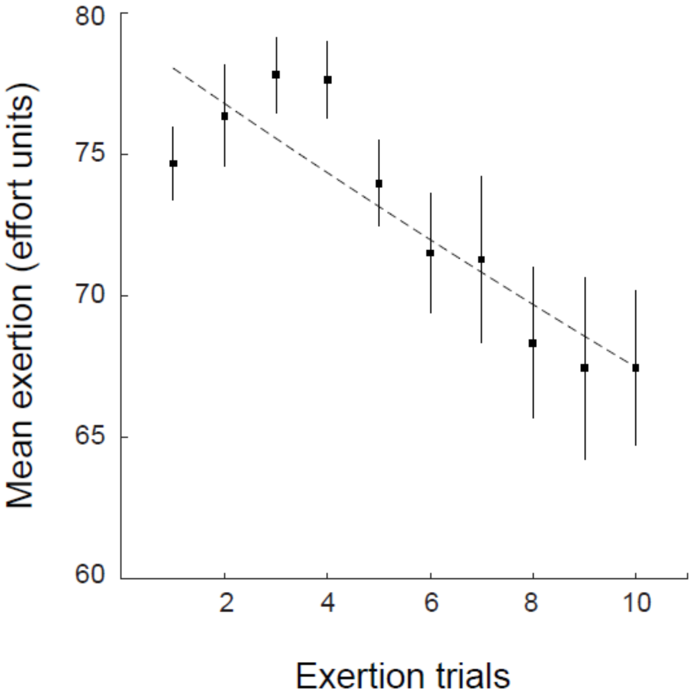

Supplementary Figure 4 (n = 20).

Illustration of the group-level mean exertion decay across all participants over the course of the first ten exertion trials, within the first exertion block of the Fatigue Choice Phase. The dashed line represents the results of the exponential decay model fit to the group-level means. Error bars indicate SEM.

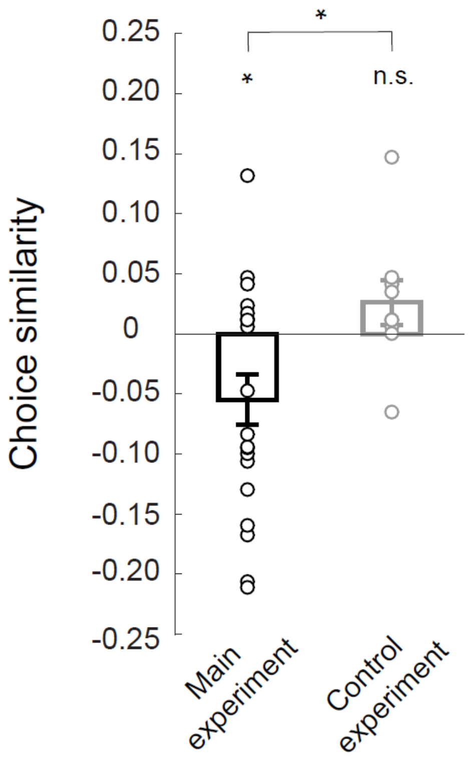

Supplementary Figure 5 (Participants from the Main Experiment and Control Experiment 2; n = 29).

Comparison of choice similarity metrics (see Supplementary Methods for details) between the main fatigue experiment and control experiment 1, averaged across choices. Relative to the Baseline Choice Phase, more positive values indicate more risk-seeking behavior in the second choice phase, whereas negative values indicate more risk-averse behavior. Participants' choice behavior in the control experiment was not significantly different comparing the first and second choice phases (two-tailed one-sample t_8 = 1.40, p = 0.20), whereas choice behavior between the Baseline and Fatigue Choice Phase was significantly different (two-tailed one-sample t_{19} = -2.59, p = 0.02), consistent with our observed fatigue-induced increase in effort subjectivity parameter ρ. The results of a two-tailed two-sample t-test comparing these choice similarity metrics, between experimental

groups indicates that these effects are significantly different ($t_{27} = 2.38$, $p = 0.02$), supporting the idea that the observed change in choice behavior in our main experiment is not merely an effect of choice exposure alone. Error bars indicate SEM. *$p < 0.05$.

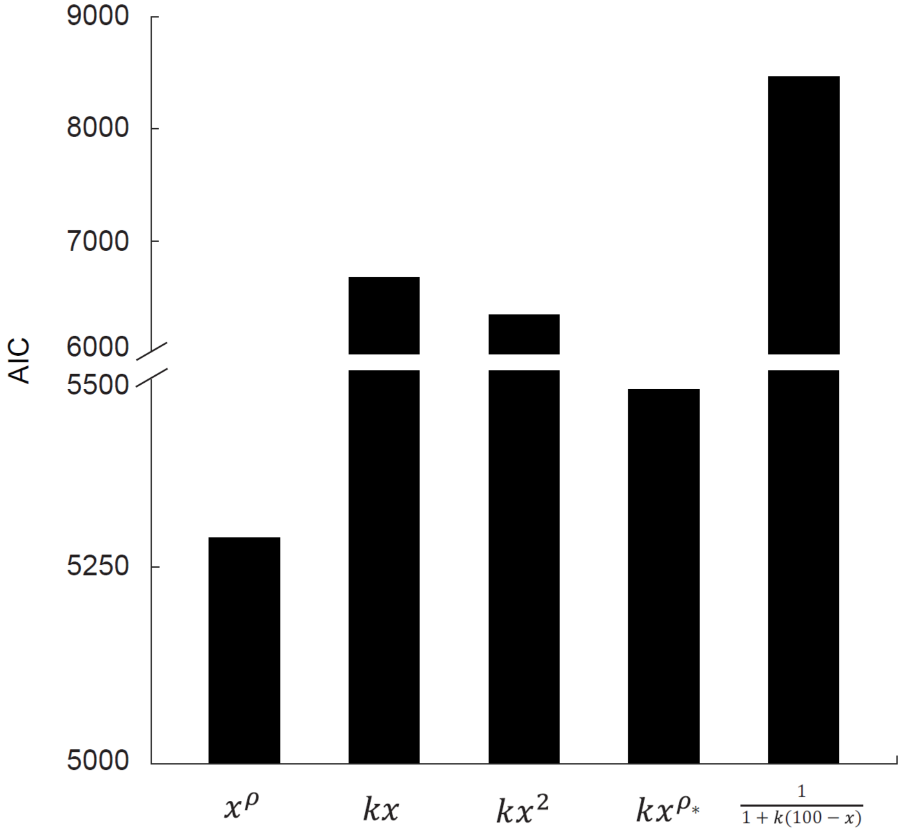

Supplementary Figure 6 (n = 20).

Model comparison of different effort utility functions. We performed a series of maximum likelihood estimations using effort utility models that have been implemented in previous studies of effort-based decision-making (Klein-Flügge et al. 2016; Chong et al. 2017; Hogan et al. 2018), to assess which utility function best captured fatigue-induced changes in subjective effort valuation. Group-level AIC measures are shown above. Lower AIC measures indicate a more descriptive model. The utility function x^{ρ} best described the choice data across conditions. It should be noted that the difference in group-level AIC between the two best models (x^{ρ}, $kx^{\rho *}$) was 189.7. Details of this analysis are described in the Supplementary Experimental Methods section.

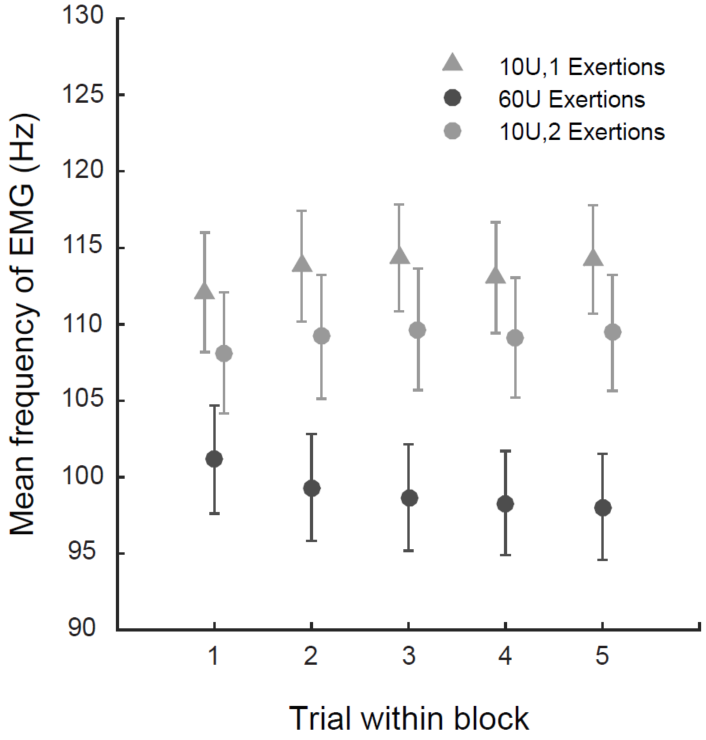

Supplementary Figure 7 (Control Experiment 2; n = 17).

Mean frequency of the power spectrum of EMGs on a trial-by-trial basis across sections of the Modified Fatigue Choice Phase. Decreases in the mean frequency of the power spectrum of the EMG signal are physiological reflections of muscle fatigue. There was a significant decrease in mean frequency of EMGs over trials (within block) for the 60U exertion section. Error bars indicate SEM.

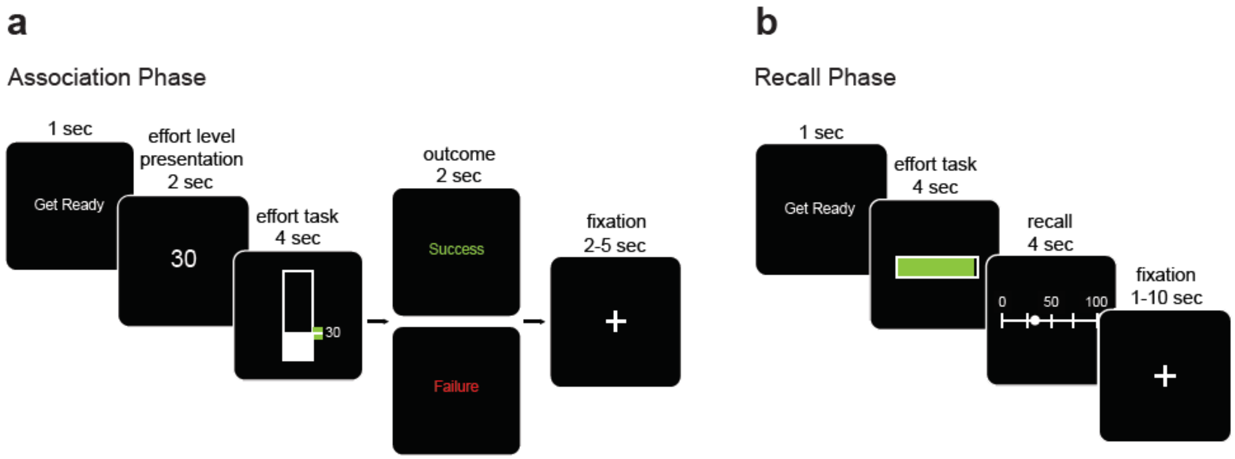

Supplementary Figure 8.

a, Association Phase; participants were trained to associate numeric effort levels with force exerted on a hand-clench dynamometer. Effort levels ranged from 0 (no force) to 100 (80% of maximum grip force). A training block consisted of five trials each at a series of target effort levels. Each trial began with presentation of the numeric target, followed by an effortful grip with real-time visual feedback of the exerted force represented as a bar that increased in height with increased exertion. A green visual cue was also displayed, within which participants were instructed to maintain their exerted effort. Feedback of success or failure was provided at the end of each trial.

b, Recall Phase; participants were instructed to fill a horizontal bar by gripping the transducer. On each trial, the full bar corresponded to a different target effort level that was unknown to participants. Successfully achieving the target effort resulted in the bar turning from red to green. Following exertion, participants used a mouse to select a value along a 0-100 number line to indicate the effort level they believed they had squeezed. No feedback was provided as to the accuracy of participants' reported effort levels.

SUPPLEMENTARY TABLES

Brain region	Laterality	Peak MNI coordinates (mm)			Peak t value
		x	y	z	
Anterior cingulate	L	-8	24	42	4.63
Dorsomedial prefrontal cortex	R	16	36	48	4.16
Insula	R	34	20	2	4.07
Insula	L	-32	24	2	3.21
Premotor cortex	R	38	14	54	4.20
Cerebellum	R	12	-32	-18	3.83
Midbrain	R	8	-10	-6	3.78
Parietal cortex	R	50	-36	42	3.43
Supplementary motor cortex	R	14	16	54	3.17

Supplementary Table 1. Brain regions with a significant increase in fMRI BOLD signal for the difference between chosen and unchosen effort value at the time of choice, across both the Baseline and Fatigue Choice Phases ($p < 0.005$, uncorrected).

Brain region	Laterality	Peak MNI coordinates (mm)			Peak *t* value
		x	y	z	
Parietal cortex	R	48	-36	44	4.15
Temporal cortex	R	58	-34	-16	3.86
Occipital cortex	R	32	-62	20	4.64
Insula	R	34	18	2	3.97
Supplementary motor cortex	R	6	16	56	3.77
Cingulate cortex	R	26	-38	32	3.72
Anterior cingulate	L	-14	40	0	3.70
Ventrolateral prefrontal cortex	R	34	40	2	3.60
Cerebellum	L	-16	-26	-30	3.60

Supplementary Table 2. Brain regions with a significant increase in fMRI BOLD signal for the difference between chosen and unchosen effort value at the time of choice, between the Baseline and Fatigue Choice Phases (Fatigue – Baseline) ($p < 0.005$, uncorrected).

		Peak MNI coordinates (mm)			
Brain region	Laterality	x	y	z	Peak *t* value
Caudate	L	-16	26	4	4.57
Caudate	R	12	24	4	4.08
Dorsolateral prefrontal cortex	R	30	40	42	4.85
Dorsolateral prefrontal cortex	L	-46	2	6	4.00
Temporal cortex	R	50	-46	16	4.70
Temporal cortex	L	-54	-50	16	4.09
Supplementary motor cortex	R	10	4	50	4.34
Supplementary motor cortex	L	-6	-8	54	3.34
Parietal cortex	R	26	-46	62	4.15
Parietal cortex	L	-58	-32	26	4.10
Cingulate cortex	L	-30	-60	16	10.16
Parahippocampal gyrus	R	38	-46	2	9.77
Occipital cortex	L	-24	-88	-4	7.14
Entorhinal cortex	R	20	2	-28	4.87
Amygdala	R	24	-10	-10	4.61
Premotor cortex	R	24	-6	66	4.42
Anterior cingulate	R	22	-12	34	3.95
Insula	R	28	26	2	3.87
Cerebellum	L	-36	-44	-28	3.79
Primary somatosensory cortex	R	50	-22	30	3.71
Orbitofrontal cortex	R	6	50	-12	3.62

Supplementary Table 3. Brain regions with a significant decrease in fMRI BOLD signal following fatiguing exertions at the time of choice (irrespective of effort value), between the Baseline and Fatigue Choice Phases ($p < 0.005$, uncorrected).

Brain region	Laterality	Peak MNI coordinates (mm) x	y	z	Peak *t* value
Cerebellum	R	26	-80	-14	4.85
Cerebellum	L	-14	-64	-18	3.41
Anterior cingulate	R	18	0	44	6.50
Anterior cingulate	L	-8	12	34	3.69
Dorsolateral prefrontal cortex	R	22	44	18	3.84
Dorsolateral prefrontal cortex	L	-28	16	34	3.25
Premotor cortex	L	-32	-14	58	4.25
Ventromedial prefrontal cortex	R	14	56	-12	4.01
Ventromedial prefrontal cortex	L	-10	52	-6	3.44
Precuneus	L	-14	-70	38	3.51
Precuneus	R	2	-74	34	3.08
Occipital cortex	L	-32	-82	26	3.40
Parietal cortex	L	-58	-30	22	3.26
Dorsomedial prefrontal cortex	L	-14	60	24	3.23
Thalamus	R	14	-4	8	3.70
Temporal cortex	L	-38	-42	-10	3.35
Retrosplenial cortex	L	-8	-46	24	3.29
Insula	R	40	2	14	3.27
Entorhinal cortex	L	-18	10	-26	3.20

Supplementary Table 4. Brain regions that arise from entering the change in effort subjectivity $\rho_{Fatigue} - \rho_{Baseline}$ as a covariate for the decreasing activity between the Fatigue and Baseline Choice Phases (p < 0.005, uncorrected).

SUPPLEMENTARY METHODS

Subjective Effort Function Model Comparison

To assess alternative effort utility functions that might capture participants' choice data, across both the Baseline and Fatigue Choice Phases, we performed a supplementary analysis in which we re-estimated effort subjectivity parameters using different cost functions. We assumed a participant's cost function $V(x)$ for effort x had one of the forms:

	Cost Function: $V(x)$	Estimated Parameters
(1)	x^{ρ}	$\tau_{Baseline}$, $\tau_{Fatigue}$, $\rho_{Baseline}$, $\rho_{Fatigue}$
(2)	kx	$\tau_{Baseline}$, $\tau_{Fatigue}$, $k_{Baseline}$, $k_{Fatigue}$
(3)	kx^2	$\tau_{Baseline}$, $\tau_{Fatigue}$, $k_{Baseline}$, $k_{Fatigue}$
(4)	kx^{ρ}	$\tau_{Baseline}$, $\tau_{Fatigue}$, $k_{Baseline}$, $k_{Fatigue}$, ρ*
(5)	$1/(1+k(100+x))$	$\tau_{Baseline}$, $\tau_{Fatigue}$, $k_{Baseline}$, $k_{Fatigue}$

*In Model 4, a single parameter ρ is modeled across both the Baseline and Fatigue Choice Phases. This assumes a constant marginal sensitivity of effort that does not change as a result of fatigue.

The effort level x is defined as negative, considering force production is perceived as a loss. τ represents a participant's choice consistency, and ρ and k capture aspects of participants' subjective valuations of effort.

Model 1 was informed by our previous study of effort valuation (Hogan et al. 2018), and was used for the main effort utility analyses in the manuscript. This model assumes that fatigue changes the marginal utility of effort.

Models 2 through 4 are variants of models proposed in previous works (Klein-Flügge et al. 2016; Chong et al. 2017; Hogan et al. 2018). These models include a parameter k that

represents a rescaling of effort value. Rather than fatigue acting on effort valuation through changes in marginal utility alone, it could modify subjective valuation by inflating the cost of effort (e.g., 60 units of effort is rescaled to 80 units of effort when fatigued). We tested the possibility of fatigue's influence on effort valuation through a model considering effort rescaling alone (Model 2), and assuming a fixed quadratic relationship across the Baseline and Fatigue Choice Phases (Model 3). We also tested models that included representations of both marginal utility and rescaling. Model 4 assumed a constant marginal utility of effort across the Baseline and Fatigue Choice Phases, as well as a rescaling of effort value between states.

Model 5 used a hyperbolic formulation to capture changes in subjective effort value between the Baseline and Fatigue Choice Phases.

Representing the effort levels as prospective costs, and assuming participants combine probabilities and utilities linearly, the relative value between the two effort options can be written for model *J* as follows:

$$RV_{sure,J}(G,S) = Value_J(sure) - Value_J(gamble)$$

$$RV_{sure,J}(G,S) = V_J(S) - 0.5 * V_J(G)$$

$RV_{sure,J}$ denotes the difference in value between the two options for model *J*. *G* represents the effort prospect for the "Flip" option, *S* represents the effort prospect listed for the "Sure" option, and both $G < 0$ and $S < 0$ for all trials.

The probability that a participant chooses the sure option for the kth trial is given by the softmax function:

$$P_{k,J}\left(RV_{sure,J}(G,S)\right) = 1/[1 + \exp\left(-\tau RV_{sure,J}(G,S)\right)]$$

Where τ is a non-negative temperature parameter representing the stochasticity of a participant's choice (τ = 0 corresponds to random choice).

We used maximum likelihood to estimate the relevant parameters for each participant, using the 170 trials of effort choices (G,S) from the Baseline Choice Phase, and the 170 trials of effort choices (G,S) from the Fatigue Choice Phase (removing missed trials from both phases), with a participant's choice denoted by $y \in \{0,1\}$. Here, y = 1 indicates that the participant chose the "Sure" option. This estimation was performed by maximizing the likelihood function separately for each participant, with choice data across both the Baseline and Fatigue Choice Phases:

$$\sum_{k=1}^{340} y_i \log\left(P_k(G,S)\right) + (1 - y_i)\log\left(1 - P_k(G,S)\right)$$

Though choices from both phases used same formulation of $RV_{sure,J}$ defined above, some models required the estimation of separate parameters for both the Baseline and Fatigue Choice Phases (e.g. $\rho_{Baseline}$ and $\rho_{Fatigue}$). In the estimator, this was accomplished by

denoting separate parameters used in the probability softmax function for each phase, though both contributed to the likelihood estimation being maximized.

SUPPLEMENTARY NOTES

- Statistically testing the effect in Supplementary Figure 7, a general linear mixed effects model revealed a significant decrease of mean frequency of EMGs over trials (within block) for the 60U exertion section (fixed effect β_{trial} = -0.75 (SE = 0.14), p < 0.001).

- A subset of the alternative models presented in Supplementary Figure 6 (described in the Supplementary Materials) tested the possibility that fatigue-induced changes in effort-based decision-making were the result of a linear rescaling of effort value caused by fatigue. In essence, fatigue could reduce an individual's maximum exertion capacity and inflate effort values separately from effects associated with changes in marginal utility of effort (e.g., 60 units of effort in a baseline/rested state could be rescaled to 80 units of effort in a fatigued state). Our model comparisons showed that utility functions that represented such a fatigue-induced rescaling did not provide the best description of our choice data. Together these results suggest that the influence of fatigue on effort-based choice is best represented through changes in marginal utility of effort.

- An important factor influencing valuation of effort is the context in which fatiguing physical exertions are experienced. In this study we showed that mere exposure to exertion was not enough to elicit changes in effort preferences, and when controlling for the number of exertions, participants' effort preferences were modulated by the magnitude of the fatiguing exertions. However, physically fatiguing tasks are often

associated with contextual components that could modulate the subjective value of physical effort. For example, the experience of running a distance race involves exerting a great deal of physical effort, similar to training, however there is added cognitive effort associated with race strategy that could make race performance particularly effortful. There are any number of factors (e.g., boredom, task-framing, social context) that may influence how physical efforts are experienced, and these factors may inflate or diminish the subjective value of physical effort. Investigating the role of contextual experience on effort valuation and fatigue will be an important future direction in understanding how individuals generate a subjective value of effort.
