# Supplementary material for: Neural mechanisms underlying the effects of physical fatigue on effort-based choice
Source: Nat Commun. 2020 Aug 12;11:4026. doi: 10.1038/s41467-020-17855-5 (PMC7424567; doi:10.1038/s41467-020-17855-5)
Supplement: Supplementary file 3 — Reporting Summary [file 41467_2020_17855_MOESM3_ESM.pdf]

nature research

Corresponding author(s): Vikram S. Chib

Last updated by author(s): June 19, 2020

Reporting Summary

Nature Research wishes to improve the reproducibility of the work that we publish. This form provides structure for consistency and transparency in reporting. For further information on Nature Research policies, see Authors & Referees and the Editorial Policy Checklist.

Statistics

For all statistical analyses, confirm that the following items are present in the figure legend, table legend, main text, or Methods section.

n/a	Confirmed	
☐	☒	The exact sample size (n) for each experimental group/condition, given as a discrete number and unit of measurement
☐	☒	A statement on whether measurements were taken from distinct samples or whether the same sample was measured repeatedly
☐	☒	The statistical test(s) used AND whether they are one- or two-sided<br>*Only common tests should be described solely by name; describe more complex techniques in the Methods section.*
☐	☒	A description of all covariates tested
☐	☒	A description of any assumptions or corrections, such as tests of normality and adjustment for multiple comparisons
☐	☒	A full description of the statistical parameters including central tendency (e.g. means) or other basic estimates (e.g. regression coefficient) AND variation (e.g. standard deviation) or associated estimates of uncertainty (e.g. confidence intervals)
☐	☒	For null hypothesis testing, the test statistic (e.g. F, t, r) with confidence intervals, effect sizes, degrees of freedom and P value noted<br>*Give P values as exact values whenever suitable.*
☐	☒	For Bayesian analysis, information on the choice of priors and Markov chain Monte Carlo settings
☒	☐	For hierarchical and complex designs, identification of the appropriate level for tests and full reporting of outcomes
☐	☒	Estimates of effect sizes (e.g. Cohen's d, Pearson's r), indicating how they were calculated

Our web collection on statistics for biologists contains articles on many of the points above.

Software and code

Policy information about availability of computer code

Data collection	The experimental task was programmed in Matlab 2013b using Psychtoolbox-3 (version 3.0.11).
Data analysis	Behavioral and fMRI ROI data were analyzed in Matlab 2015a. To extract subjective effort parameters maximum likelihood estimation was performed using the fminsearch function.<br><br>fMRI data were analyzed using SPM 12 (v6225) and custom scripts in MATLAB. Moderation analyses were carried out using the glmfit function in Matlab.<br><br>Parameter estimation in the Control Experiment 2 was implemented using Monte-Carlo Markov Chain sampling methods provided by Stan version 2.19 and implementing a similar methodology as described by the hBayesDM package.

For manuscripts utilizing custom algorithms or software that are central to the research but not yet described in published literature, software must be made available to editors/reviewers. We strongly encourage code deposition in a community repository (e.g. GitHub). See the Nature Research guidelines for submitting code & software for further information.

Data

Policy information about availability of data

All manuscripts must include a data availability statement. This statement should provide the following information, where applicable:

- Accession codes, unique identifiers, or web links for publicly available datasets
- A list of figures that have associated raw data
- A description of any restrictions on data availability

The source data underlying Figures 2a-e, 3c, 4b, d-f , 5c, d, 6a-e, 7a-c and Supplementary Figs. 1–7; and the imaging contrasts for Figures 3a,b and 4a,c are available for download at https://osf.io/w2rdm/. Fully anonymized raw behavioral and neuroimaging data files are available from the corresponding author upon reasonable request.

Field-specific reporting

Please select the one below that is the best fit for your research. If you are not sure, read the appropriate sections before making your selection.

- [ ] Life sciences
- [x] Behavioural & social sciences
- [ ] Ecological, evolutionary & environmental sciences

For a reference copy of the document with all sections, see nature.com/documents/nr-reporting-summary-flat.pdf

Behavioural & social sciences study design

All studies must disclose on these points even when the disclosure is negative.

Study description

This study investigated the influence of physical fatigue on valuation of effort. Quantitative data were acquired, including behavioral variables, fMRI measures, and EMG.

Research sample

Participants were recruited from the Johns Hopkins university and Medical Instititute community. All participants were right-handed and prescreened to exclude those with prior history of neurological or psychiatric illness. A total of 30 particpants took part in the study and the the final analysis for the primary experiment included N = 20 participants in total (mean age, 24 years; age range, 18-34 years; 9 females).

A group of 10 healthy participants, separate from those that performed the main experiment, took part in the first control experiment. One participant was excluded because they were unable to generate salient associations between effort levels and applied effort. The final analysis included N = 9 participants in total (mean age, 19 years; age range, 18-21 years; 6 females).

A group of 21 healthy right-handed participants, separate from those in either of the previous two experiments, took part in the sectond control experiment. Two participants were unable to complete the experiment after exceeding the specified failure threshold, and were therefore not considered for analysis. Of the remaining participants, one was excluded because they did not generate a salient association between effort levels and applied effort. The final analysis for this experiment included a total of N = 17 participants (mean age, 26 years; age range, 21-37 years; 11 females).

Sampling strategy

Participants took part in the experiment on a first-come, first-served basis with considerations for individual schedules and MRI booking constraints.

This sample size was motivated based on a previous studies of effort-based choices by our group:

Hogan PS, Galaro JK, Chib VS. Roles of Ventromedial Prefrontal Cortex and Anterior Cingulate in Subjective Valuation of Prospective Effort. Cerebral Cortex, 29(10), October 2019, p. 4277–4290, https://doi.org/10.1093/cercor/bhy310.

Data collection

The experimenter was present throughout every step of data collection, and at least one MRI Technician was also present during functional imaging sessions. The experimenter was not blinded to the experiment conditions or hypotheses.

A 3 Tesla Philips Achieva Quasar X-series MRI scanner and radio frequency coil was used for all the MR scanning sessions. High resolution structural images were collected using a standard MPRAGE pulse sequence, providing full brain coverage at a resolution of 1 mm x 1 mm x 1 mm. Functional images were collected at an angle of 30° from the anterior commissure-posterior commissure (AC-PC) axis, which reduced signal dropout in the orbitofrontal cortex. Forty-eight slices were acquired at a resolution of 3 mm x 3 mm x 2 mm, providing whole brain coverage. An echo-planar imaging (FE EPI) pulse sequence was used (TR = 2800 ms, TE = 30 ms, FOV = 240, flip angle = 70°).

Presentation of visual stimuli and acquisition of behavioral data were achieved using custom MATLAB (http://www.mathworks.com) scripts implementing the PsychToolBox libraries. During functional magnetic resonance imaging (fMRI), visual feedback was presented via a projector positioned at the back of the room. Participants viewed a reflection of the projector in a mirror attached to the scanner head coil.

An MRI compatible hand clench dynamometer (TSD121B-MRI, BIOPAC Systems, Inc., Goleta, CA) was used to record grip force effort exertion. During experiments, signals from this sensor were sent to our custom designed software for visual real-time feedback of participants' exertion. Effort exertion was performed while participants held the force transducer in their right hand with arm extended while lying in the supine position.

To record participants' choices we used an MRI compatible multiple button-press response box (Cedrus RB-830, Cedrus Corp., San Pedro, CA) held in the left hand.

To record electromyogram (EMG) signals during the control experiment all exertion trials (for MVC, Association, Recall, and Modified Fatigue Choice Phases), we examined muscle activations using surface electromyograms (EMGs). Three disposable electrodes (NeuroPlusTM A10040 Electrodes; Vermed.com, Buffalo, NY) recorded muscle activity targeting the right flexor digitorum superficialis muscle. EMG signals were amplified (AMT-8; Bortec Biomedical Ltd., Calgary, Alberta, Canada) and bandpass filtered with high- and low-pass cutoff frequencies of 10 and 1000 Hz, and additionally filtered with a 60 Hz notch-filter. Signals were sampled at 5 kHz by a 16-bit data acquisition system (CED Micro1401-3; Cambridge Electronic Design Ltd., Cambridge, England).

Timing

2016/05/01 - 2019/11/01

Data exclusions

For the primary experiment: 10 participants were ultimately excluded from the final analyses for one or a combination of pre-established behavioral reasons. Participants were excluded if they were unable to generate salient associations between effort levels and applied

effort (n = 5; r-squared between reported and actual effort during the Recall Phase, was less than 0.5). Additionally, participants were excluded if their subjectivity parameter ρ, obtained from either Choice Phase (Baseline or Fatigue), was beyond two standard deviations of the population mean for that phase and their temperature parameters (τ) were near zero, indicative of random choice (n = 5).

For the first control experiment: 3 participants were excluded for one or a combination of pre-established behavioral reasons. Participants were excluded if they were unable to generate salient associations between effort levels and applied effort (n = 1; r-squared value between reported effort during the Recall Phase and perfect reporting was less than 0.5) or if poor estimates were obtained for the subjectivity parameters (n = 2).

For the second control experiment: Two participants were unable to complete the experiment after exceeding the specified failure threshold (see below), and were therefore not considered for analysis. Of the remaining participants, one was excluded because they did not generate a salient association between effort levels and applied effort (r-squared between reported and actual effort during the Recall Phase was less than 0.5); another was excluded because their percentage of accepted effort gambles during the Baseline Choice Phase, was beyond two standard deviations of the mean proportion of acceptance. These exclusion criteria were established based the previous experiments.

Non-participation	No participant dropped out/declined participation.
Randomization	Participants were not allocated into experimental groups.

Reporting for specific materials, systems and methods

We require information from authors about some types of materials, experimental systems and methods used in many studies. Here, indicate whether each material, system or method listed is relevant to your study. If you are not sure if a list item applies to your research, read the appropriate section before selecting a response.

Materials & experimental systems

n/a	Involved in the study	
☒	☐	Antibodies
☒	☐	Eukaryotic cell lines
☒	☐	Palaeontology
☒	☐	Animals and other organisms
☐	☒	Human research participants
☒	☐	Clinical data

Methods

n/a	Involved in the study	
☒	☐	ChIP-seq
☒	☐	Flow cytometry
☐	☒	MRI-based neuroimaging

Human research participants

Policy information about studies involving human research participants

Population characteristics	See above.
Recruitment	Participants were recruited via both local recruitment postings as well as an online, institution-wide announcement system – both of which provided rudimentary information about the study as well as appropriate means of contacting the experimenters about potential involvement.
Ethics oversight	Johns Hopkins School of Medicine Institutional Review Board

Note that full information on the approval of the study protocol must also be provided in the manuscript.

Magnetic resonance imaging

Experimental design

Design type	Task-based fMRI; block-design.
Design specifications	Imaging data was collected when participants made effort decisions. There were a total of 170 choice trials in the Baseline Choice Phase and 170 choice trials in the Fatigue Choice Phase. The Baseline Choice Phase was separated into 2 scanning sessions, each lasting approximately 12 minutes. During the Fatigue Choice phase, after an initial block of ~10 exertion trials, participants made 10 choice trials followed by ~5 trials of exertion. This sequence was repeated 17 times. The Fatigue Choice Phase was separated into 3 scanning sessions, each lasting approximately 12 minutes.
Behavioral performance measures	During choice we recorded participants' responses (acceptance/rejection of options) and response times. During exertion trials we recorded participants' applied forces. We modeled choice trials in which participants didn't not respond as missed trials . To ensure participants were properly performing the choice task we evaluated the variability in participants' choice, as captured by our choice models (details in the Methods Section). To ensure that participants were exerting effort we evaluated the mean exertion during exertion trials to ensure they were reaching the target effort levels.

Acquisition

Imaging type(s)	Functional
Field strength	3T
Sequence & imaging parameters	Functional images were collected at an angle of 30° from the anterior commissure-posterior commissure (AC-PC) axis, which reduced signal dropout in the orbitofrontal cortex. Forty-eight slices were acquired at a resolution of 3 mm x 3 mm x 2 mm, providing whole brain coverage. An echo-planar imaging (FE EPI) pulse sequence was used (TR = 2800 ms, TE = 30 ms, FOV = 240, flip angle = 70°).
Area of acquisition	Whole Brain

Diffusion MRI ☐ Used ☒ Not used

Preprocessing

Preprocessing software	The SPM12 (6225) spatial and temporal preprocessing functionality was utilized for preprocessing of the functional imaging data. Slice-timing correction, cross-temporal realignment, spatial normalization, and smoothing were all accomplished within the SPM12 framework. Parameters of interest for these preprocessing modules are as follows:<br><br>Realignment:<br>Smoothing (FWHM): 5 mm kernel<br>Two-pass procedure, aligning images to mean of images after first alignment<br><br>Normalization:<br>Bias regularization: 0.0001<br>Bias FWHM: 60 mm cutoff<br><br>Smoothing:<br>FWHM: 8 mm kernel
Normalization	Spatial normalization was accomplished within the SPM12 framework, using a segmentation routine which incorporates tissue probability maps into the normalization/registration process.
Normalization template	Tissue probability atlas/maps: TPM.nii probabilistic atlass of the brain and cervical spinal cord (provided within SPM12 framework; by Claudia Blaiotta, Patrick Freund, M. Jorge Cardoso, and John Ashburner)<br>Affine regularization: ICBM space template for European brains (provided within SPM12 framework)
Noise and artifact removal	To account for participant motion in the SPM GLMs, regressors modeling the head motion as derived from the affine part of the realignment procedure were included in the model.
Volume censoring	We did not utilize any volume censoring procedure in our image pre-processing.

Statistical modeling & inference

Model type and settings	Univariate models were used. A general linear model (GLM) was used to estimate participant-specific (first-level), voxel-wise, statistical parametric maps (SPMs) from the fMRI data. The GLM included categorical regressors beginning at the time of trial presentation and ending when a choice was indicated, for both the Baseline and Fatigue Phase, for the chosen and unchosen effort options. Each of these categorical regressors included unorthogonalized parametric modulators corresponding to the expected value of the risky ('Flip') and sure options. Trials with missing responses were modeled as a separate nuisance regressors. The Fatigue Choice Phase included an additional nuisance regressor, modeled as a 4 second block, corresponding to the exertion trials between choice blocks. Finally, regressors modeling the head motion as derived from the affine part of the realignment procedure were included in the model.
Effect(s) tested	With the first-level models, we created group models (second-level) to test brain areas that were generally sensitive to chosen effort value. This was done by creating contrasts with the aforementioned parametric modulators for chosen and unchosen effort values, at the time of choice (i.e., difference between the value of the chosen and unchosen options, across both the Baseline and Fatigue Phases). We also tested for regions of the brain in which effort value was sensitive to changes in bodily state induced by fatigue (i.e., difference between the value of the chosen and unchosen options, between the Baseline and Fatigue Phases). Additionally, we tested for changes in regions of the brain that were more generally sensitive to changes in bodily state induced by fatigue by examining the difference between the Baseline and Fatigue Choice Phases, regardless of the effort values in question.

Specify type of analysis: ☐ Whole brain ☐ ROI-based ☒ Both

Anatomical location(s)	We analyzed brain signals related to chosen effort value within independent regions of interest (ROIs) taken at peak coordinates from Neurosynth.org when using the term "effort": rInsula MNI coordinates (x, y, z) = [36, 24, 0]; lInsula MNI coordinates (x, y, z) = [-36, 24, 0]; anterior cingulate cortex (x, y, z) = [0, 14, 48]. To analyze motor signals related to fatigue we used coordinates for premotor cortex reported in an independent study of fatiguing physical grip exertion (dorsal premotor cortex: (x, y, z) = [-36, -14, 64]).<br><br>To clarify the signal patterns in our contrasts we created plots of effect sizes at the peak of activity (Figures 3c, 4b). It is important to note that these signals are not statistically independent and these plots were not used for statistical inference, but rather shown solely for illustrative purposes. Statistical inference was carried out within the SPM framework by small volume correcting in 5 mm spheres within our a priori coordinates.<br><br>Motor area results for the PPI analysis are reported within a 5 mm spherical ROI centered on coordinates from Figure 4a. Effect sizes in all voxels within this sphere were averaged to obtain an overall effect size for the PM ROI.
Statistic type for inference (See Eklund et al. 2016)	Voxel-wise.
Correction	FWE correction was utilized as reported in the manuscript and figure legends.

Models & analysis

n/a	Involved in the study
☒	☐ Functional and/or effective connectivity
☒	☐ Graph analysis
☒	☐ Multivariate modeling or predictive analysis
